# Identification of promising anti-EBOV inhibitors: *de novo* drug design, molecular docking and molecular dynamics studies

**DOI:** 10.1098/rsos.220369

**Published:** 2022-09-28

**Authors:** Eslam A. R. Mohamed, Sayed F. Abdelwahab, Ahmad M. Alqaisi, Amaal Mohammed Salih Nasr, Heba Ali Hassan

**Affiliations:** ^1^ Department of Chemistry, Faculty of Science, Minia University, Minia 61511, Egypt; ^2^ Department of Pharmaceutics and Industrial Pharmacy, College of Pharmacy, Taif University, PO Box 11099, Taif 21944, Saudi Arabia; ^3^ Chemistry Department, University of Jordan, Amman 11942, Jordan; ^4^ Department of Chemistry, Faculty of Science, Universiti Putra Malaysia, 43400 Serdang, Malaysia; ^5^ Department of Pharmacognosy, Faculty of Pharmacy, Sohag University, Sohag 82524, Egypt

**Keywords:** deep learning, LigDream, Ebola virus, molecular docking, molecular dynamics

## Abstract

The Ebola virus (EBOV) outbreak was recorded as the largest in history and caused many fatalities. As seen in previous studies, drug repurposing and database filtration were the two major pathways to searching for potent compounds against EBOV. In this study, a deep learning (DL) approach via the LigDream tool was employed to obtain novel and effective anti-EBOV inhibitors. Based on the galidesivir (BCX4430) chemical structure, 100 compounds were collected and inspected using various *in silico* approaches. Results from the molecular docking study indicated that mol1_069 and mol1_092 were the best two potent compounds with a docking score of −7.1 kcal mol^−1^ and −7.0 kcal mol^−1^, respectively. Molecular dynamics simulations, in addition to binding energy calculations, were conducted over 100 ns. Both compounds exhibited lower binding energies than BCX4430. Furthermore, compared with BCX4430 (%Absorption = 60.6%), mol1_069 and mol1_092 scored higher values of % Absorption equal to 68.1% and 63.7%, respectively. The current data point to the importance of using DL in the drug design process instead of conventional methods such as drug repurposing or database filtration. In conclusion, mol1_069 and mol1_092 are promising anti-EBOV drug candidates that require further *in vitro* and *in vivo* investigations.

## 1. Introduction

**Figure 1.**
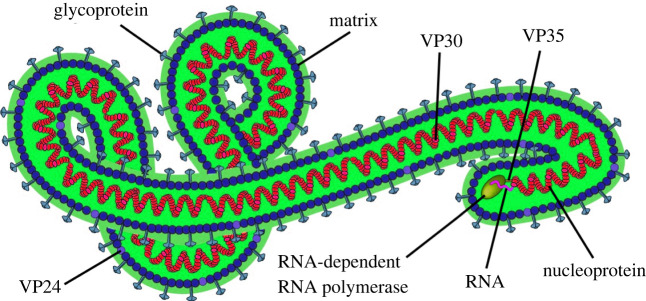
Structure of Ebola virus virion.

In 2014, the Ebola virus disease (EVD) emerged and was characterized by a high mortality rate in both humans and non-human primates [1,2]. The Ebola virus (EBOV) belongs to the family *Filoviridae* and the order *Mononegavirales* [3]. A total of five EBOV species were identified as the major causative agents of EVD, namely *Sudan*, *Zaire*, *Tai Forest*, *Reston* and *Bundibugyo* [4]. Of these five species, *Zaire ebolavirus* has been identified as the most prevalent and virulent with a mortality rate of 90% and causing dramatic damage in many countries [5]. Common symptoms of EVD involve eye inflammation, hemorrhagic rash, fever and a marked decrease in plasma volume [6]. EBOV genomic RNA encodes seven drug targets, including viral proteins (VPs), RNA-dependent RNA polymerase (L), nucleoprotein (NP) and glycoprotein (GP) [7,8]. Viral proteins include VP24, VP30, VP35 and VP40. EBOV is enveloped by a membrane taken from an infected cell and studded with glycoproteins. A layer of matrix proteins supports the membrane on the inside and holds a cylindrical nucleocapsid (NC) at the centre, which stores and delivers the RNA genome. Figure 1 illustrates the position of each protein around the EBOV membrane.

Based on previous studies, VP24 was found to be the most important protein for EBOV because of its role in viral transcription, replication and maturation of NCs [9]. In addition to the aforementioned functions, VP24 binds to karyopherin alpha, which blocks the accumulation of tyrosine-phosphorylated STAT1 (pSTAT) in the nucleus [10,11]. Accordingly, VP24 protein was selected as a suitable drug target to be inhibited to block EVD. Conventional methods in the drug discovery and development process can take several years, and the cost of bringing a novel drug to market can be around two billion dollars [12]. Instead of all these efforts, computer-aided drug design (CADD) can be an effective, rapid and cost-efficient method to design novel drugs [13,14]. In the course of searching for standard VP24 inhibitors, galidesivir (BCX4430) was chosen as a result of its role in blocking viral replication [15–17].

As a replacement for old-fashioned approaches to discover new inhibitors, the deep learning (DL) method has been successfully applied to *de novo* drug design. DL can be readily defined as an evolution and extension of machine learning (ML). In practical terms, DL is just a part of ML and mainly relies on artificial neural networks. The major difference between DL and ML is that the latter uses algorithms developed to execute well-defined tasks. DL is characterized by a data representation based on multiple layers of a matrix, where each layer takes the output of the previous layer as input. Both ML and DL are important subfields of artificial intelligence (AI).

Explainable artificial intelligence (XAI) or interpretable machine learning (IML) has recently been developed to improve the transparency, explainability and interoperability of DL models [18]. XAI is a set of strategies and techniques that enable human users to understand and rely on the findings produced by ML algorithms [19]. It displays the expected influence and potential biases of the AI algorithms, particularly in the field of drug discovery [20]. Therefore, XAI can improve the characterization and understanding of the produced results and output values when employing DL models in *de novo* drug design [21].

In order to achieve the above purpose, the LigDream tool (https://playmolecule.org/LigDream/) [22] was used to design novel compounds based on the initial structure of BCX4430. The LigDream tool was exploited to generate new functional groups and scaffolds attributed to the starting structure of the chosen molecule by long short-term memory (LSTM) [23]. In the field of drug design, OnionNet [24] and Pafnucy [25] are well-known examples of the use of neural networks in predicting protein–ligand binding affinity. One hundred new BCX4430 derivatives have been designed with different features and functional groups from the parent compound. In the current study, a molecular docking, as well as molecular dynamics (MD), study was executed to explore the effectiveness of the generated molecules. To get a deeper insight into the potency of the studied inhibitors, drug-likeness analysis was performed to check the bioavailability of the best-ranked drugs. All results of both molecular docking and drug-likeness analyses of the studied inhibitors were compared with the parent molecule (BCX4430). The current study demonstrates the importance of using ML, in particular DL, in the process of computational drug design. The current data will be the cornerstone for future *in vivo* and *in vitro* studies to ensure the efficacy of the identified anti-EBOV^VP24^ inhibitors.

## 2. Computational methodology

### 2.1. Protein preparation

By searching via the RCSB Protein Data Bank, two PDB IDs were identified for Zaire EBOV^VP24^ namely 4M0Q and 4U2X. As a consequence of the resolutions of both protein X-ray structures, 4M0Q (1.92 Å) was selected instead of 4U2X (3.15 Å). 4M0Q exists in a dimeric conformation consisting of two chains (A/B) and both are completely identical. The three-dimensional crystal structure of 4M0Q was obtained (chain: A) [26] with an experimental data snapshot; method: X-ray diffraction, R-Value Free: 0.23, and R-Value Work: 0.209. All crystallographic water molecules, ions, extracellular domains and heteroatoms were ignored. Modeller software was exploited to build all missing residues [27]. The protonation state of EBOV^VP24^ amino acids was adjusted by using the H++ web-based server (http://biophysics.cs.vt.edu/H++). Also, missing hydrogen atoms were successfully added [28,29]. The physical conditions of external dielectric = 80, salinity = 0.15, internal dielectric = 10 and pH = 6.5 were set to investigate the pKa of 230 residues.

### 2.2. Active site characterization

**Figure 2.**
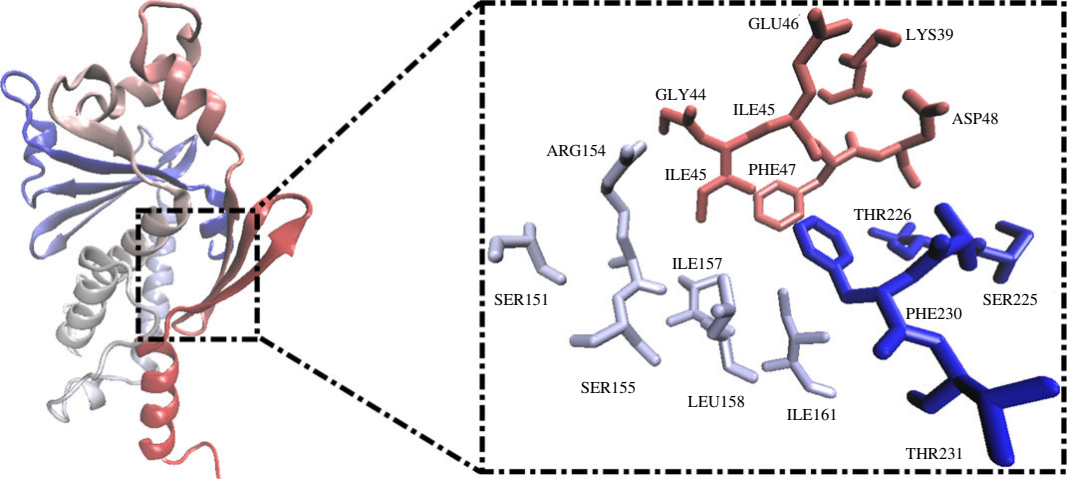
Representation of predicted binding site residues using the CASTp tool.

For identification of the EBOV^VP24^ active site residues, Computed Atlas of Structure Surface Topography of proteins (CASTp) was employed (http://www.sts.bioe.uic.edu/castp/) [30,31]. Sites without mouth openings were eliminated, which includes small volumes and areas. Figure 2 illustrates the binding pocket of EBOV^VP24^ and the names of the selected amino acids. The results obtained are compatible with the previous studies that used other bioinformatics tools [32].

### 2.3. Generation of novel lead compounds

BCX4430 is an adenosine nucleoside analogue that plays an important role as an antiviral drug ‘specifically' against EBOV. The IUPAC nomenclature of BCX4430 is (2S,3S,4R,5R)-2-(4-amino-5H-pyrrolo[3,2-d]pyrimidin-7-yl)-5-(hydroxymethyl)pyrrolidine-3,4-diol, indicating the position, number and direction of the existing functional groups. As can be observed from the BCX4430 chemical structure, three rings with different attached groups and linkages are present. For further explanation, the three rings have been named as A, B and C. Ring A is a six-membered ring structure with a free −NH2 group. Ring B is a five-membered ring structure with secondary amine (−NH−). Ring C is a five-membered ring structure with three hydroxyl groups (−OH). Undoubtedly, groups such as −NH2, −NH− and −OH will participate in the interaction with EBOV^VP24^, mainly through hydrogen bonding and other different interactions. On this basis, unconventional ideas were developed to find novel inhibitors with high efficiency towards EVD. For this purpose, generative shape-based neural network decoding named the LigDream module [22] of the PlayMolecules server (https://playmolecule.org/LigDream/) was used. The SMILE string of the parent molecule (BCX4430) was uploaded to the LigDream server and computed to design 100 new SMILE strings for different molecules. Structures of the 100 compounds are illustrated in the electronic supplementary material, figure S1. Captioning and auto-encoders are the major networks used because of their ability to design several molecules starting with one reference structure. Using Chem3D Pro 12.0 software, the three-dimensional structure of molecules was sketched and followed by energy minimization via the MM2 force field.

### 2.4. Molecular docking analysis

A molecular docking study was carried out to investigate the potency of the studied inhibitors. AutoDock Vina [33] was the software of choice due to its accuracy and numerous features. Parameters were set to 25 Å × 25 Å × 25 Å for the grid box and 1.0 Å for the spacing centre. Default values of the docking parameters were used except for the exhaustiveness parameter that was set to 200. Different poses were inspected (nine poses for each inhibitor) and the best interaction energy was selected. Based on the results of the molecular docking study, the best compounds were considered for further analysis of molecular features. Results and protein−ligand interactions were analysed using BIOVIA discovery studio visualizer [34].

### 2.5. Molecular dynamics simulations

As a conclusion of the molecular docking study, mol1_069 and mol1_092 were selected and subjected to 100 ns MD simulation using the web-based CHARMM-GUI [35–37] interface with the CHARMM36 force field [38]. The whole simulations were performed following the NAMD 2.13 [39] package. Solvation of protein–ligand complexes was done within a cubic box of the transferable intermolecular potential with a three-points (TIP3P) explicit solvation model [40]. Periodic boundary conditions were adjusted with a dimension of 115.2 Å^3^. Applied parameters were successfully produced employing the CHARMM General Force Field (CGenFF) [41]. Neutralization of total protein−ligand complexes was performed via satisfactory numbers of the Na+/Cl− ions with a concentration equal to 0.9% (physiological solution). Minimization, equilibration and production were included in the MD protocol used. Following system preparation, 5000 step energy minimizations were performed using a combination of steepest and conjugate gradient algorithms to clear any inappropriate geometries or steric clashes. Simulation time of 2 fs was applied for all MD simulations. Then, equilibration was done in the canonical (NVT) ensemble. Finally, the isothermal–isobaric (NPT) ensemble was specified for the production step. Over the 100 ns MD simulation, the Nosé–Hoover Langevin piston barostat [42] was used to maintain the pressure at 1 atm, with a Langevin piston decay of 0.05 ps and a period of 0.1 ps. Moreover, the Langevin thermostat [43] was employed to adjust the temperature to 298.15 K. The cutoff distance was set to 12.0 Å for the short-range non-bonded interactions in compliance with a pair list distance of 16 Å. Parameters of Lennard−Jones interactions were set to 8.0 Å. Furthermore, the particle-mesh Ewald (PME) method [44,45] was employed to assign the long-range electrostatic interactions. For all simulation cells, 1.0 Å grid spacing was established. The SHAKE algorithm [46] was used to constrain all covalent bonds including hydrogen atoms. Aiming to achieve consistency, the same protocol was used for all MD simulations.

### 2.6. Binding energy calculations

To calculate the relative free energies of binding of the most promising compounds complexed with EBOV^VP24^, the molecular mechanics-generalized Born surface area (MM/GBSA) approach [47,48] was used, carried out in the MOLAICAL code [49]. The MM/GBSA (*ΔG*_binding_) energy was estimated as follows:

2.1 $$\Delta G_{\mathrm{binding}}=\;\Delta G_{C}-\Delta G_{P}-\;\Delta G_{L},$$

where the ligand (*L*) binds to the protein receptor (*R*) to form complex (*C*).

This can be represented by contributions of various interactions,

2.2 $$\Delta G_{\mathrm{binding}}=\Delta H-T\Delta S=\;\Delta E_{MM}+\Delta G_{\mathrm{Sol}}-\;T\Delta S.$$

The above terms (Δ*E*_MM_), (Δ*G*_Sol_) and (*T*Δ*S*) represent the gas phase molecular mechanics change, the solvation Gibbs energy and the conformational entropy, respectively. For more explanation, Δ*E*_MM_ can be calculated as the totality of changes in the electrostatic energies Δ*E*_ele_ added to the van der Waals energies Δ*E*_vdW_ and the internal energies Δ*E*_int_. Furthermore, the term Δ*G*_Sol_ can be estimated as the total value of polar solvation, non-polar solvent and *T*Δ*S*. Calculation of the polar and non-polar solvation can be performed using the generalized Born model and the solvent-accessible surface area (SASA), respectively. Also, TΔS can be calculated employing normal mode analysis. In MM/GBSA calculations, the values of 78.5 and 0.03012 kJ mol^−1^ Å^2^ were assigned for the solvent dielectric constant and the surface tension constant, respectively.

### 2.7. Computational analysis of molecular features

*In silico* properties such as drug-likeness, physico-chemical properties and medicinal chemistry were explored to verify the activity of the studied compounds towards EBOV^VP24^. The SwissADME server (http://www.swissadme.ch/) [50] was employed to run all types of *in silico* analyses. Features such as Lipinski's (Pfizer) rule of five [51], Ghose (Amgen) [52], Veber (GSK) [53], Egan (Pharmacia) [54] and Muegge (Bayer) [55] were inspected to get a deep insight into the drug-likeness of inspected compounds. Also, the bioavailability score was considered [56]. In terms of the physico-chemical properties, the following criteria were examined: topological-polar surface area (TPSA), number of hydrogen bond donors (nOHNH), number of hydrogen bond acceptors (nON), number of rotatable bonds (Nrotb), molecular weight (MWt) and percentage of absorption (%ABS) [57]. Also, Brenk alerts [58], pan-assay interference (PAINS) [59], leadlikeness [60] and synthetic accessibility were tested to determine the validity of the studied compounds for medicinal chemistry. In order to detect any undesirable toxic properties of the designed compounds, the web-server ProTox-II (http://tox.charite.de/protox_II/) [61] was employed. ProTox-II uses molecular similarity and ML models to detect toxicity endpoints such as hepatotoxicity, cytotoxicity, carcinogenicity, immunotoxicity and mutagenicity, as well as adverse outcome pathways (AOPs) (Tox21). ProTox-II predicts toxicity primarily based on functional group similarity between the molecules under investigation and the *in vivo* cases (e.g. hepatotoxicity, carcinogenicity), as well as *in vitro* assays (e.g. Tox21 assays, hepG2 cytotoxicity assays, Ames bacterial mutation assays, immunotoxicity assays) included in the database. The ProTox-II prediction is mainly based on the Tox21 (Toxicology in the 21st Century) platform [62,63] and focused on two major groups of AOPs: the nuclear receptor pathway (NRP) and the stress response pathway (SRP). There are seven target–pathway-based models related to NRP: androgen receptor (AR), androgen receptor ligand binding domain (AR-LBD), aryl hydrogen receptor (AhR), oestrogen receptor alpha (ER), aromatase, peroxisome proliferator-activated receptor gamma (PPARGamma) and oestrogen receptor ligand binding domain (ER-LBD). Also, there are five target–pathway-based models related to SRP: nuclear factors (erythroid-derived 2)-like 2/antioxidant responsive element (ARE), mitochondrial membrane potential (MMP), heat shock factor response element (HSE), ATPase family AAA domain-containing protein 5 (ATAD5) and phosphoprotein tumor suppressor (p53). Only the two-dimensional chemical structure or SMILES can be used as an input, and a report containing the toxicity profile is appropriately generated.

## 3. Results and discussion

Advances in the field of computational chemistry made it possible to test compounds with the advantage of rapid testing and accurate results. The procedure of molecular docking analysis is a computer-aided foretelling of the drug efficacy that uses specified algorithms to estimate the favourable ligand orientation in the binding site. In addition, computational studies can be helpful in the drug filtration process and identification of the most potent inhibitors that can be used in further *in vitro* experiments.

### 3.1. Molecular docking study

Using AutoDock Vina, molecular docking calculations were performed for 100 compounds to identify the efficiency and potency of the studied compounds as anti-EBOV^VP24^. Molecular docking is considered to be one of the most favourable approaches in computational chemistry to provide reliable results about how effective a drug truly is and whether it might be valid [64].

**Figure 3.**
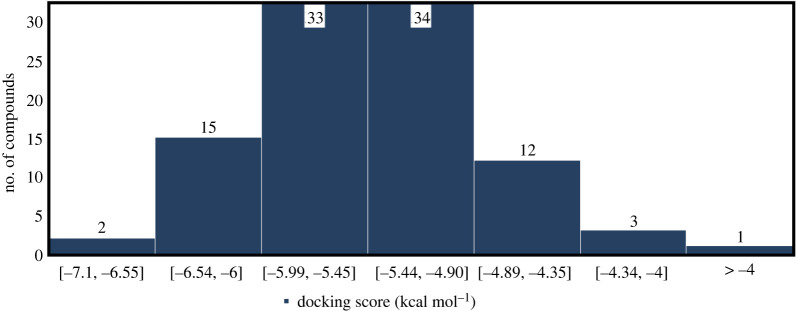
Relationship between docking score (kcal mol^−1^) and the number of compounds.

The binding energy, also known as ligand binding affinity, can be described as the decrease in total complex energy when a drug is linked to the target protein. According to the docking scores, compounds were ranked and compared with BCX4430. Results are outlined in the electronic supplementary material, table S1 and a statistical representation can be found in figure 3. From the data in figure 3, it is interesting that only one compound obtained a docking score higher than −4.0 kcal mol^−1^. More than half of the investigated compounds (67%) achieved docking scores in the range of –4.9 to –5.99 kcal mol^−1^. Of the total, 17 compounds scored binding affinities equal to or lower than that obtained by the reference molecule BCX4430 (greater than or equal to −6.0 kcal mol^−1^). Results of the docking score for nine conformations of the best 10 compounds are summarized in the electronic supplementary material, table S2. The best binding energies were found to be for mol1_069 (−7.1 kcal mol^−1^) and mol1_092 (−7.0 kcal mol^−1^). The current analysis was performed for the conformation which had a lower binding affinity compared with the other eight conformations.

**Figure 4.**
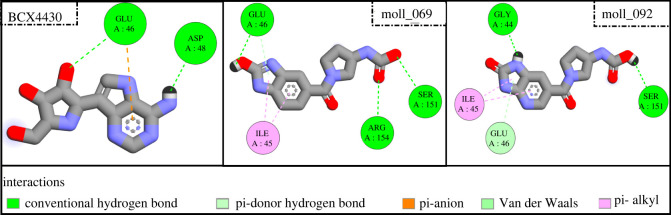
Three-dimensional representation of binding modes for the best two inhibitors and the reference molecule BCX4430.

To understand the estimated binding energies, figure 4 shows the protein–ligand interactions of the two best compounds, in addition to BCX4430. Through analysing the protein−ligand complexes in figure 4, interactions such as hydrogen bonding, π-alkyl, π-anion and van der Waals mainly contribute to the binding affinity values of the studied compounds. Herein, BCX4430 has two types of interactions with active site residues: two conventional hydrogen bonds and one π-anion interaction. On the other hand, mol1_69 exhibits three types of interactions: conventional hydrogen bonding, π-alkyl bonding and π-donor hydrogen bonding. Also, mol1_092 exhibits the same types of interactions as mol1_069. In order to get a deeper insight into all types of interactions in protein−ligand complexes, the binding features of the two best compounds are listed in table 1.

**Table 1.** Calculated molecular docking scores (in kcal mol^−1^) and binding features for BCX4430, mol1_069 and mol1_092.

molecule	docking score (kcal mol^−1^)	binding features (hydrogen bond length in ångström)
mol1_069	−7.1	GLU46 (2.87 Å), SER151 (3.08 Å), ARG154 (3.23 Å)
mol1_092	−7.0	GLY44 (2.12 Å), SER151 (2.51 Å)
BCX4430	−6.0	GLU46 (3.08 Å), ASP48 (2.13 Å)

What is interesting about the data in table 1 is that the interaction between BCX4430 and EBOV^VP24^ was through two hydrogen bonds (GLU46 and ASP48) with bond distances of 3.08 Å and 2.13 Å, respectively. There is a well-known concept that distance and hydrogen bond numbers are the two main factors that affect the binding affinity of any compound [65,66]. Accordingly, mol1_069 and mol1_092 were found to have strong hydrogen bonds, which are shorter and more prevalent than BCX4430 possesses. Three hydrogen bonds were formed with active site residues in the case of mol1_069: GLU46 (2.87 Å), SER151 (3.08 Å) and ARG154 (3.23 Å).

**Figure 5.**
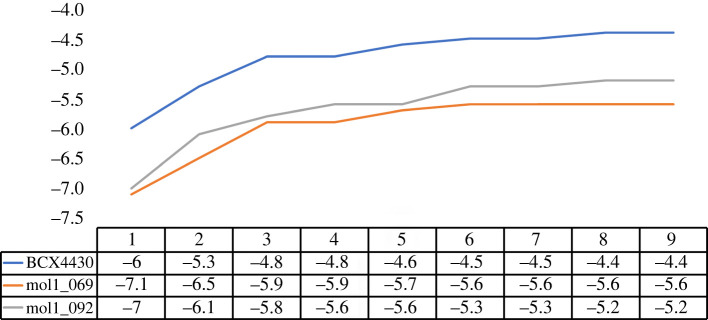
Nine poses of molecular docking for the best two compounds and BCX4430.

Repetition of GLU46 in both interactions of BCX4430 and mol1_069 with EBOV^VP24^ was noticed but with different hydrogen bond distances. In the case of the third compound (mol1_092), two hydrogen bonds with GLY44 (2.12 Å) and SER151 (2.51 Å) were constituted with the protein target. As a result of analysis of the protein–ligand interaction, it could be contemplated that GLY44, GLU46, ASP48, SER151 and ARG154 are favourable residues in the EBOV^VP24^ binding pocket. Mol1_069 and mol1_092 had the lowest docking scores in all nine poses (figure 5).

Finally, the best two compounds, mol1_069 and mol1_092, were selected for further analyses such as MD simulations, drug-likeness, physico-chemical properties and medicinal chemistry.

### 3.2. Molecular dynamics simulations

In computer-aided drug discovery, MD simulations are considered an important step in understanding interactions of protein−ligand complexes. Moreover, they provide detailed information about the binding affinity of the complex structure. As a consequence of molecular docking analysis, only two compounds (mol1_069 and mol1_092) had a docking score equal to or lower than −7.0 kcal mol^−1^. An MD simulation was run out over 50 ns and followed by 100 ns. An MM/GBSA approach was established as one of the best ways to assign the numerical values of binding affinity over the simulation process. As illustrated in table 2, both mol1_069 and mol1_092 exhibited lower binding affinities (kJ mol^−1^) over different simulation times (50 and 100 ns).

**Table 2.** The calculated MM/GBSA binding energies for mol1_069, mol1_092 and the reference molecule BCX4430.

molecule	estimated MM/GBSA binding energy (kJ mol^−1^)	
	50 ns	100 ns
mol1_069	−64.85 ± 0.84	−57.95 ± 0.780
mol1_092	−42.228 ± 0.89	−22.34 ± 0.829
BCX4430	−9.024 ± 0.60	−8.39 ± 0.459

**Figure 6.**
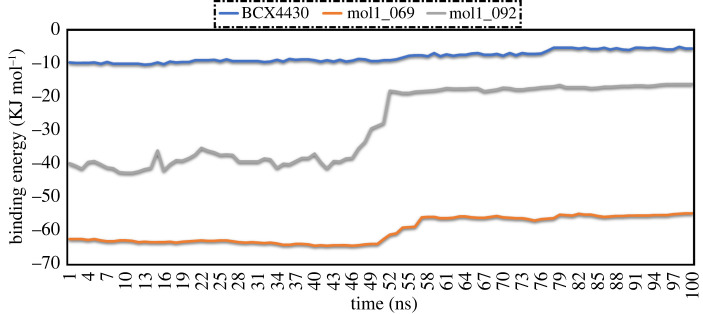
Evaluated binding energies per frame for BCX4430, mol1_069 and mol1_092 towards EBOVVP24 over 100 ns MD simulations.

Over 100 ns MD simulations, the correlation between computed binding energies and time for the mol1_069/EBOV^VP24^ and mol1_092/EBOV^VP24^ complexes was evaluated and compared with that of BCX4430 to study the inhibitor's stability inside the EBOV^VP24^ active site (figure 6). From data in figure 6, overall stabilities were noted for mol1_069 and BCX4430 inside the EBOV^VP24^ active site through 100 ns MD simulations. On the other hand, mol1_092 showed relative instability during the first stage of MD simulations.

**Figure 7.**
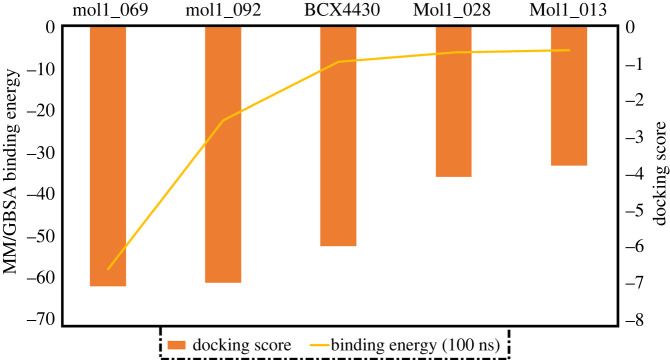
MM/GBSA binding energy and docking scores of BCX4430, the highest two compounds (mol1_069 and mol1_092) and the lowest two compounds (mol1_028 and mol1_013).

Figure 7 depicts docking scores for BCX4430, the top-ranked compounds and the lowest-ranked compounds, as well as their estimated MM/GBSA binding energy over 100 ns. The docking score ranking is in agreement with binding energy rankings for the studied compounds. Mol1_069 obtained the highest docking score and also the highest binding energy in the EBOV^VP24^ active site. Furthermore, mol1_028 and mol1_013 exhibited lower binding energies towards the EBOV^VP24^ active site, which corresponded to docking scores.

### 3.3. Post-dynamics analyses

**Figure 8.**
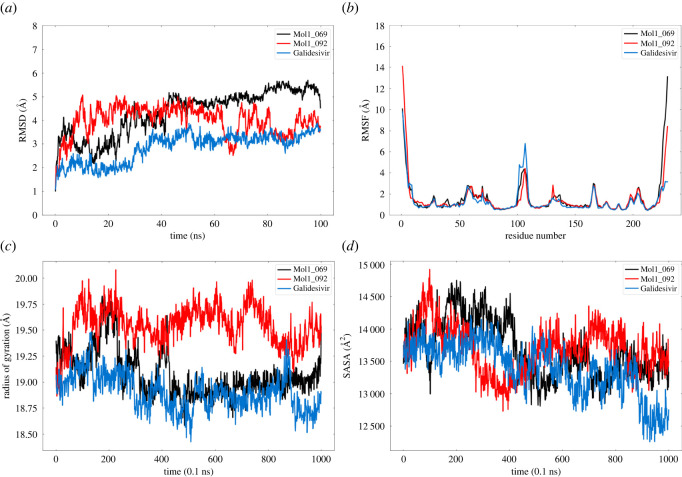
Analysis of molecular dynamics simulation trajectories of protein–ligand complexes over 100 ns simulation. (a) root-mean-square deviation (RMSD), (b) root-mean-square fluctuation (RMSF), (c) radius of gyration (Rg) and (d) solvent-accessible surface area (SASA).

After conducting measurements of the deviation of coordinates concerning a reference structure, the root-mean-square deviation (RMSD) was inspected. RMSD results indicate the overall stability of the system. Herein, RMSD analysis of the best two compounds and the reference molecule was executed over 100 ns. Results are illustrated in figure 8*a*. From figure 8*a*, it can be seen that mol1_069 and BCX4430 exhibited noticeable stability through the 100 ns MD simulation. The following equation can be used to calculate RMSD:

3.1 $$\mathrm{RMSD}=\sqrt{\frac{1}{N}\;\;\;m_{i}\overset{N}{\underset{i=1}{\sum }}{\left|r_{\mathrm{final}}(i)-r_{\mathrm{initial}}\;(i)\right|}^{2}\;\;},$$

where *N*, *m_i_*, *r*_final_ (*i*) and *r*_initial_ (*i*) represent the number of atoms, mass of atom (i), coordinates of the atom (i) at its final state and coordinates of the atom (i) at its initial state, respectively.

Another type of analysis called root-mean-square fluctuation (RMSF) was measured to assess the dynamic behaviour of protein residues with respect to their reference position over the simulation time. In other words, RMSF analysis can be useful in determining the conformational changes in the protein structure as a result of its binding with the ligand. Low values of RMSF can be found in the regions of helix and sheets, which represent the rigid structures in protein.

On the other hand, regions containing sheets and turns exhibit greater fluctuations. Data of the RMSF analysis are graphed in figure 8*b*. Unlike BCX4430, normal fluctuations were observed for mol1_092 and mol1_069 that complexed with a protein receptor. RMSF can be estimated using the following equation:

3.2 $$\mathrm{RMSF}=\sqrt{\frac{1}{T}\;\;\;\overset{T}{\underset{i=1}{\sum }}{\left|r_{\mathrm{final}}(i)-r_{\mathrm{initial}}\;(i)\right|}^{2}\;\;},$$

where *T* represents the simulation time.

What is noticed from the equations of RMSF and RMSD is that the former is averaged over time, determining the value of each particle *i*, but RMSD is estimated over the particles, specifying time detailed values.

Furthermore, analysis of the radius of gyration (Rg) was implemented to investigate the compactness as well as the protein molecules' size. As a result of Rg analysis, the stability of protein−ligand complexes can be assessed. The results are shown in figure 8*c*, in which it can be seen that mol1_069 and BCX4430 displayed an acceptable result in the Rg analysis.

In the current study, the SASA was calculated in the interest of characterizing the region of the protein that is exposed enough to interact with the nearby solvent molecules. SASA can be regarded as a specifying factor for protein folding and stability. Data are illustrated in figure 8*d*. Compared with BCX4430, both mol1_069 and mol1_092 exhibited relative stability.

### 3.4. Computational analysis of molecular features

In the current research, drug-likeness analyses were performed to investigate whether the inhibitors under study could be valid as drug-like compounds or not. If so, their chances of becoming drug-like molecules improve. Various rules such as Lipinski, Ghose, Veber, Egan and Muegge were explored to assess the drug-like property of the best two compounds and BCX4430. Mol1_092 and mol1_069 scored no violations of any rules. On the other hand, BCX4430 scored one violation each for Lipinski (NHorOH > 5), Ghose (WLOGP < −0.4), Veber (TPSA > 140) and Egan (TPSA > 131.6), and two violations for Muegge (XLOGP3<−2, H-don > 5). The bioavailability score gives an approximate prediction of measurable Caco-2 permeability or the oral bioavailability rate (at least 10%). The bioavailability score depends mainly on TPSA, total charge and violation of the Lipinski rule. Testified compounds can be classified based on four probabilities of 11%, 17%, 56% or 85%. Drug-likeness violations are summarized in table 3. The best two compounds and also BCX4430 scored the same bioavailability score (55%). Mol1_069 exhibited zero Lipinski, Ghose, Veber, Egan and Muegge violations.

**Table 3.** Drug-likeness for the highest ranked compounds and the standard molecule BCX4430.

molecule	drug-likeness violations					
	Lipinski violations	Ghose violations	Veber violations	Egan violations	Muegge violations	bioavailability score
mol1_069	0	0	0	0	0	0.55
mol1_092	0	1	0	0	0	0.55
BCX4430	1	1	1	1	2	0.55

To get a deeper insight into the molecular features of the studied compounds, physico-chemical properties were also explored (table 4). The TPSA can readily be defined as the surface sum for all polar atoms, in particular nitrogen and oxygen. There is an inverse relationship between TPSA and the permeability or bioavailability of compounds [67]. Values of TPSA for mol1_069 and mol1_092 were 118.55 Å and 131.18 Å, respectively, which is less than for BCX4430 (140.31 Å). According to the results of TPSA, mol1_069 and mol1_092 are better able to cross the blood–brain barrier (BBB) compared with BCX4430. Based on the TPSA, the percentage of absorption (%ABS) can be calculated [57] as follows: %ABS = 109−[0.345 * TPSA]. Owing to the low TPSA, mol1_069 and mol1_092 scored high values of %ABS (68.1% and 63.7%, respectively), which is higher than for BCX4430 (60.6%). The values of %ABS indicate that the best two compounds should possess a good cellular plasmatic membrane permeability. The MWt of BCX4430 (265.27 g mol^−1^), mol1_069 (290.27 g mol^−1^), and mol1_092 (291.26 g mol^−1^) meet the Lipinski rule that MWt should be less than 500. For the best two compounds, the nOHNH and nON are less than 5 and 10, respectively. The flexibility of a molecule originates from the Nrotb that allow it to freely rotate around itself [53]. Molecular flexibility represents the ease of the molecule transversing around the membrane. The optimum Nrotb is less than 8 [68]. Mol1_069 and mol1_092 have the same Nrotb (5), which is less than for BCX4430 (6).

**Table 4.** Physico-chemical properties for the best two compounds and BCX4430.

molecule	physico-chemical properties					
	MWt (g mol^−1^)	nON	nOHNH	Nrotb	TPSA (Å)	%ABS
mol1_069	290.27	4	5	4	118.55	68.1
mol1_092	291.26	4	5	4	131.18	63.7
BCX4430	265.27	6	6	2	140.31	60.6

In medicinal chemistry (table 5), the Brenk and PAINS compounds can be used to predict toxic, reactive and unstable fragments that exist in the structure. BCX4430 and the best two compounds have zero alerts in both Brenk and PAINS descriptors. All compounds have a good indication (yes) for leadlikeness filter. Synthetic accessibility (SA) is considered the main factor in the drug selection process. SA score is primarily based on the hypothesis that the frequency of molecular fragments in available molecules correlates with the facility of synthesis. SA scores range from 1 (very easy) to 10 (very difficult). Mol1_069 scored the highest SA of 2.48. Additionally, mol1_092 scored a good value of 2.63. Compared with BCX4430 (SA = 3.48), mol1_069 and mol1_092 were found to have higher values in the SA filter.

**Table 5.** Medicinal chemistry of the reference molecule BCX4430, mol1_069 and mol1_092.

molecule	medicinal chemistry			
	PAINS (alert)	Brenk (alert)	Leadlikeness	synthetic accessibility
mol1_069	0	0	yes	2.48
mol1_092	0	0	yes	2.63
BCX4430	0	0	yes	3.48

In terms of toxicity assessment (table 6), the top two compounds and BCX4430 showed varying degrees of inactivity towards the five toxicity targets. Regarding hepatotoxicity, BCX4430 demonstrated the lowest probability (68%) compared with mol1_069 and mol1_092, which had high probabilities of 76% and 73%, respectively. Moreover, mol1_069 and mol1_092 showed substantial inactivity in the immunotoxicity assessment with probabilities of 95% and 99%, respectively. Evaluation of mutagenicity revealed that BCX4430 exhibited moderate inactivity (56%) compared with mol1_069 (68%) and mol1_092 (67%). In contrast, BCX4430 showed a high level of inactivity with regard to carcinogenicity (67%), while mol1_069 and mol1_092 exhibited moderate levels of inactivity. In terms of cytotoxicity, the three compounds displayed similar behaviours of inactivity, ranging from 60% (BCX4430) to 67% (mol1_092). The full toxicity report for each compound is illustrated in the electronic supplementary material, figure S2. Strikingly, mol1_092 and mol1_069 demonstrated greater inactivity (greater than or equal to 85%) towards all targets in both NRP and SRP. On the other hand, BCX4430 showed moderate inactivity towards ATAD5 in SRP and good inactivity towards other targets.

**Table 6.** Predicted toxicities for BCX4430, mol1_069 and mol1_092.

molecule	toxicity risks				
	hepatotoxicity	immunotoxicity	mutagenicity	carcinogenicity	cytotoxicity
mol1_069	inactive (76%)	inactive (95%)	inactive (68%)	inactive (59%)	inactive (65%)
mol1_092	inactive (73%)	inactive (99%)	inactive (67%)	inactive (57%)	inactive (67%)
BCX4430	inactive (68%)	inactive (96%)	inactive (56%)	inactive (67%)	inactive (60%)

## 4. Conclusion

The current study was set up to design novel potent inhibitors against EBOV^VP24^. Instead of conventional methods such as drug repurposing or database filtration, DL was applied as an efficient approach in the drug selection process. One hundred compounds were derived through the LigDream platform based on the chemical structure of BCX4430. The best drug candidates, namely mol1_069 and mol1_092, which displayed the lowest docking scores of −7.1 kcal mol^−1^ and −7.0 kcal mol^−1^, respectively, were selected for further inspections against EBOV^VP24^. Results of MD simulations over 100 ns declared the efficiency of both compounds. Concerning physico-chemical properties, the %ABS of mol1_069 and mol1_092 was found to be 68.1% and 63.7%, respectively. Furthermore, mol1_069 and mol1_092 scored higher values of SA (2.48 and 2.63, respectively), which means that they are somewhat easier to synthesize. Overall, both developed compounds displayed encouraging toxicity profiles. These results demonstrate the possible efficacy of DL in designing potent and novel compounds based on a parent molecule.

## Data Availability

The data are provided in the electronic supplementary material [69].
